# Effective Management of Chronic Low Back Pain in the Elderly: A One-Year Cohort Study of Oxygen–Ozone Therapy Under CT Guidance Combined with Alpha Lipoic Acid, Palmitoylethanolamide, and Myrrh

**DOI:** 10.3390/biomedicines13051250

**Published:** 2025-05-20

**Authors:** Matteo Bonetti, Michele Frigerio, Gian Maria Ottaviani, Giannantonio Pellicanò, Alessio Zambello, Mario Muto, Francesco Carinci, Federico Maffezzoni

**Affiliations:** 1 Department of Neuroradiology, Istituto Clinico Città di Brescia Hospital, Via Bartolomeo Gualla 15, 25128 Brescia, Italy; 2 Department of Emergency, Spedali Civili di Brescia Hospital, Piazzale Spedali Civili 1, 25123 Brescia, Italy; 3 Department of Neuroradiology, Careggi University Hospital—Azienda Ospedaliera Universitaria Careggi, Largo Giovanni Alessandro Brambilla 3, 50134 Florence, Italy; 4 Department of Anesthesia and Pain Therapy, Casa di Cura Borghi Hospital, Via Francesco Petrarca 33, 21020 Brebbia, Italy; 5 Department of Neuroradiology, Cardarelli Hospital, Via Antonio Cardarelli 9, 80131 Naples, Italy; 6 Department of Translational Medicine, University of Ferrara, 44121 Ferrara, Italy; crc@unife.it; 7 Poliambulatorio Specialistico Oberdan, Via Guglielmo Oberdan 126, 25128 Brescia, Italy; dott.federicomaffezzoni@gmail.com

**Keywords:** low back pain, elderly, ozone therapy, ozone, alpha-lipoic acid, palmitoylethanolamide, myrrh

## Abstract

**Background and Objective:** This observational study aimed to evaluate the clinical efficacy of combined oxygen–ozone (O_2_-O_3_) therapy under CT guidance with the oral administration of alpha-lipoic acid (ALA), palmitoylethanolamine (PEA), and myrrh in elderly patients suffering from chronic low back pain (LBP). Given the rising prevalence of degenerative spinal diseases in older adults, this study addresses the need for effective, minimally invasive treatment options. **Methods:** A total of 276 patients aged 65 to 92 years, with chronic unilateral or bilateral LBP, underwent CT-guided paravertebral infiltrations with an O_2_-O_3_ gas mixture. This treatment was complemented with a 30-day regimen of ALA (800 mg/day), PEA (600 mg/day), and myrrh (200 mg/day). Clinical outcomes were assessed at one month and one year post-treatment using the Visual Analog Scale (VAS) and the modified McNab method. **Results**: At one month, 32.94% of patients reported an excellent improvement, with the mean VAS score dropping from 8.17 to 2.81. At the one-year follow-up, 68.15% cumulatively experienced positive outcomes, with 17.78% reporting the complete resolution of pain. In this occasion, the mean VAS score was 3.57. **Conclusions:** The study demonstrates that the combination of oxygen–ozone therapy and oral ALA, PEA, and myrrh is a promising alternative for managing chronic low back pain in the elderly, leading to significant pain reduction and improved quality of life. Findings emphasize the need for further research to validate these results and explore the long-term benefits.

## 1. Introduction

The increase in the average lifespan has been accompanied by an exponential rise in degenerative spinal diseases in the elderly [1,2]. The global population of individuals aged 60 or older is expected to triple by 2050, bringing with it a series of health-related challenges associated with aging, including low back pain (LBP), which is one of the primary causes of disability in this age group [3,4]. The risk factors for LBP in the elderly are multiple and complex [5,6,7,8,9,10], and the clinical picture is often complicated by the presence of comorbidities, psychological problems, and variations in pain perception.

Most LBP cases in the elderly are non-specific and self-limiting; however, as age advances, the risk of developing specific pathologies such as osteoporosis, vertebral fractures, and spinal stenosis increases significantly. The prevalence of severe and chronic LBP tends to increase significantly with age, with incidence rates showing a progression from 3.8% in individuals aged 77–79 years to 9.7% in those aged 90–100 years [11]. Among the elderly, it is estimated that up to 80% of residents in long-term care facilities experience musculoskeletal pain, and a significant portion of these cases involve LBP [12,13].

A review of the health conditions of the elderly has shown that many factors can influence not only the onset of LBP but also its management. These include physical and psychosocial changes associated with aging, such as spinal degeneration, physical inactivity, and comorbidities, which can complicate the situation further [14]. In particular, elderly individuals with LBP are more likely to have concomitant conditions, such as hypertension and joint pain, highlighting the need for a holistic approach to treating this population [15]. The use of analgesics in older adults entails several important considerations. While these medications can provide significant pain relief, it is crucial to be mindful of potential issues associated with their use. Older individuals may present a variety of risk factors that can make their response to analgesics different from that of younger patients [1,2,3,4,5,11,12,13,14,15,16,17,18]. It is therefore essential that clinicians and researchers address the peculiarities of LBP management in the elderly, adopting a multidisciplinary approach that considers their individual characteristics and comorbidities.

Oxygen–ozone (O_2_-O_3_) therapy is a widely utilized therapeutic option, which recently gained significant recognition, being recommended for various pathologies. Over the past 30 years, this therapy has experienced significant growth, supported by numerous scientific articles that have demonstrated its effectiveness, particularly in the treatment of hernias and spine pathologies. Introduced for the first time in 1985, O_2_-O_3_ therapy has shown positive results in the treatment of low back pain, with success rates ranging from 75% to 90% [19,20,21,22,23]. Recent reports have also highlighted good outcomes for pathologies affecting the posterior compartment of the spine [24,25,26,27,28,29,30,31,32,33,34,35].

Diseases related to the posterior compartment of the spine can have multiple etiologies, including facet joint dysfunction, spondylolisthesis, spinal stenosis, radicular cysts, intra- and inter-apophyseal synovitis, and Baastrup syndrome [1,2,3,4,5,6,7,8,9,10,11,12,13,14,15]. Therefore, it is fundamental to arrive at a precise diagnosis after an accurate clinical examination, supported by appropriate diagnostic exams. The application of advanced imaging techniques, such as computed tomography (CT) and magnetic resonance imaging (MRI), facilitates the correct diagnostic evaluation of the pathology subsequently to be treated. The use of CT is also an indispensable tool in order for the therapeutic procedural steps to be carried out with maximum effectiveness, in compliance with radiation protection protocols.

The integration of advanced imaging techniques into the diagnostic and therapeutic process not only increases the accuracy of evaluations but also ensures a high standard of safety for patients undergoing O_2_-O_3_ therapy, e.g., [24,25,34].

The mechanisms of action of O_2_-O_3_ include analgesic, anti-inflammatory, and antioxidant effects that contribute to improved cellular metabolism. O_2_-O_3_ therapy stimulates the activation of antioxidant enzymes, such as superoxide dismutase and glutathione peroxidase, reducing oxidative stress and improving the oxygen supply to tissues through vasodilation and the stimulation of angiogenesis [36,37,38,39,40,41,42,43,44].

Several studies have considered the therapeutic potential of alpha-lipoic acid (ALA), palmitoylethanolamine (PEA), and myrrh when administered orally for pain management. ALA has been shown to reduce oxidative stress and prevent damage from free radicals [45,46,47,48,49,50,51,52]. PEA acts as a biological modulator that promotes the physiological responses of tissues [53,54,55]. Myrrh is characterized by a high content of bioactive furanodienes preserved through a patented extraction process [56,57,58].

Given the beneficial effects demonstrated by these substances and their low incidence of side effects [55] and their efficacy in combination of O_2_-O_3_ therapy [59,60,61], this observational study aims to evaluate the effects of these combined treatments in an elderly population affected by LBP. In this observational study, we examined a sample of 276 patients aged between 65 and 92 years to evaluate the therapeutic efficacy of intramuscular injections of a mixture of deep intramuscular paravertebral O_2_-O_3_ supplemented via the oral administration of ALA + PEA + myrrh. Clinical outcomes were evaluated one month and one year after the completion of treatment. The findings were also compared to those of a preceding study conducted on a cohort of 129 elderly patients, who were monitored at three-month and one-year intervals [29]. This prior investigation exclusively evaluated the outcomes of CT-guided O_2_-O_3_ therapy and confirmed its therapeutic efficacy for the management of low back pain within the elderly population. This investigation aims to address the need for more effective and personalized strategies for managing LBP among the elderly to improve their quality of life and reduce the associated socio-health burden.

## 2. Materials and Methods

To fulfill the aim of the study, the following inclusion criteria were employed to define the sample:

- Patients experiencing chronic low back pain for at least 12 weeks.
- Patients aged 65 years or older.
- Presence of degenerative changes in the lumbosacral spine confirmed through CT or MRI.
- Absence of non-spinal neurological conditions (e.g., confirmed diabetic neuropathy).
- Good compliance with the follow-up assessments at one month and one year.

After reviewing and signing an informed consent form, a total of 276 patients (125 men and 151 women, aged 65 to 92 years, with a mean age of 76.2 years) suffering from chronic LBP underwent deep paravertebral CT-guided infiltration of a mixture of O_2_-O_3_ gases in correspondence with the lumbosacral segment of the spine affected by the underlying pathology. All patients were instructed to discontinue any ongoing analgesic medications, including oral anticoagulants, at least three days prior to each treatment session. No patients had received steroid treatments prior to enrollment.

The study was conducted between April 2022 and November 2024 by associating for 30 days, after the O_2_-O_3_ infiltration, the combined oral treatment with ALA + PEA + myrrh, administering 2 tablets/day.

**Figure 1:**
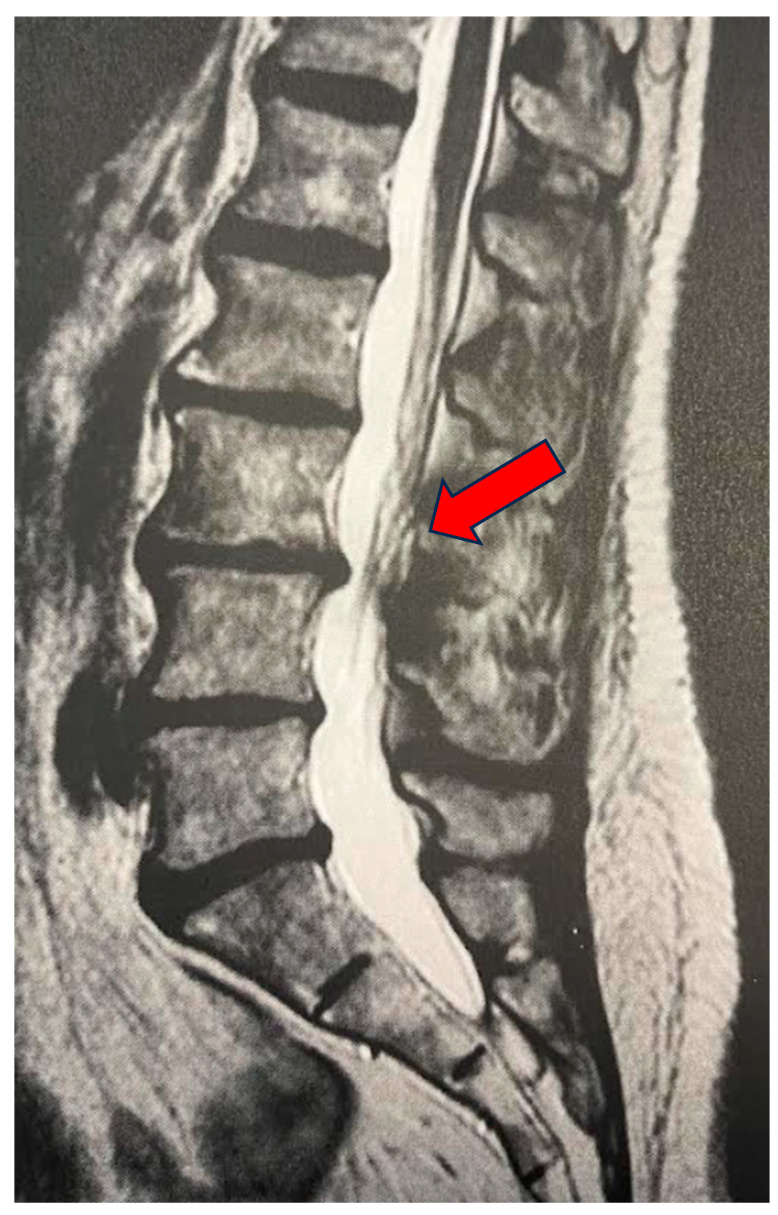
LPR of 78-year-old female. Sagittal MRI: median subligamentous disc herniation at L3–L4 (arrow). Complete resolution of low back pain symptoms at both the one-month and one-year follow-up after treatment.

**Figure 2:**
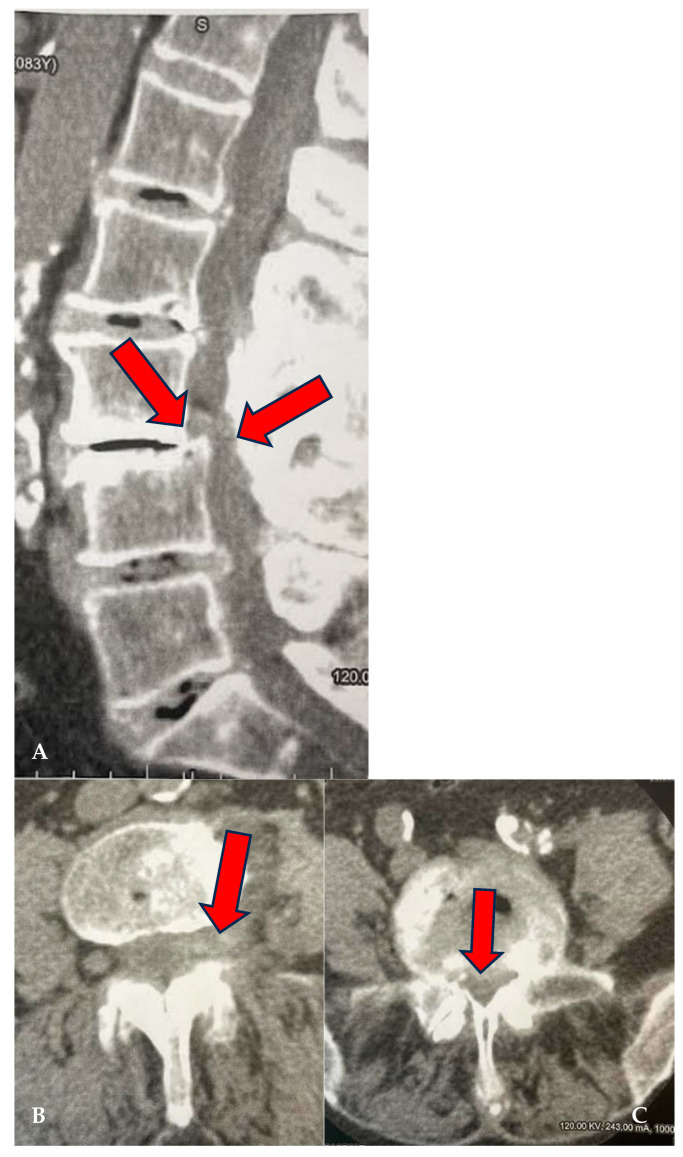
(**A**–**C**): CL of 88-year-old female treated at multiple levels (L3–L4 and L4–L5). At L3–L4, segmental canal stenosis related to pseudospondylolisthesis (**A**) (red arrows), concomitant intradiscal and foraminal left disc herniation at L3–L4 (arrow) (**B**), and circumferential disc protrusion at L4–L5 (red arrow) (**C**).

**Figure 3:**
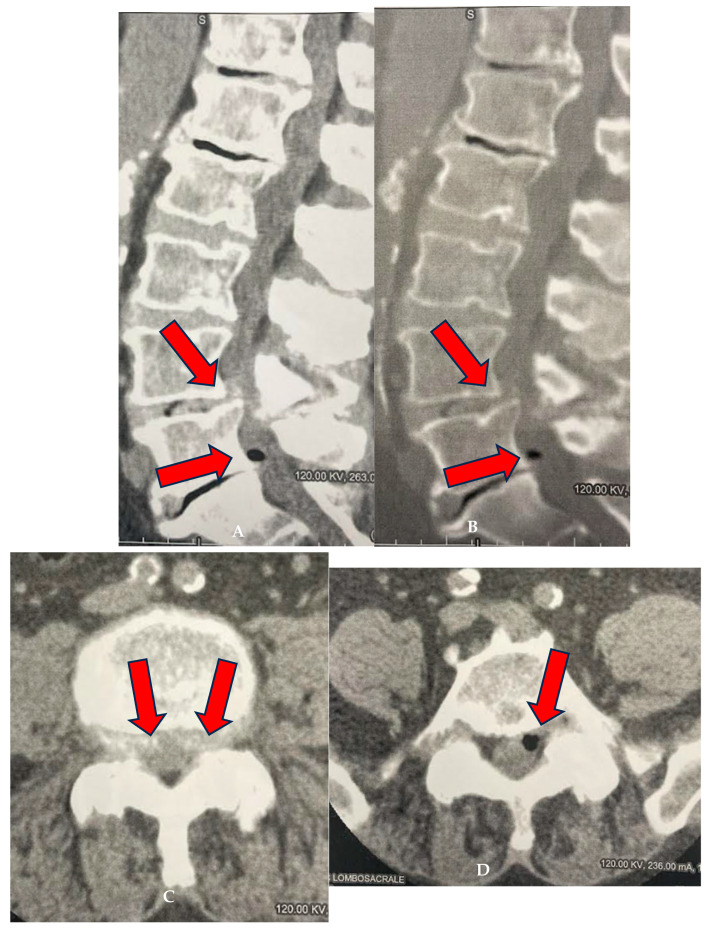
(**A**–**D**): FG of 77-year-old male treated at L4–L5 and L5–S1 levels. (**A**,**B**) Sagittal CT reconstructions using algorithms for both parenchyma and bone. L4–L5 spondylolisthesis (arrows), degenerated and thinned L5–S1 disc with vacuolated left paramedian herniation (arrow). (**C**) Axial CT scan at L4–L5: bilateral paramedian central protrusion of the annulus (arrows). (**D**) Axial scan at L5–S1: partially vacuolated left paramedian herniation causing indentation on the emergence of the left S1 nerve root (arrow).

A medical record was created for each patient at enrollment, which collected key information such as age, date of birth, date of enrollment, date of treatment, and clinical details regarding the type of pain, radiation of pain, presence of paresthesias, Lasègue sign, sensitivity levels, lower limb reflexes, and plantar and dorsal extensions of the foot, as well as dorsal extension of the great toe. The study included patients suffering from advanced degenerative zygapophyseal osteoarthritis, lumbar disc disease (including multilevel), segmental canal stenosis, and pseudospondylolisthesis, with evidence from CT or MRI (Figure 1, Figure 2 and Figure 3).

Enrolled patients presented chronic unilateral or bilateral low back pain that radiated to areas innervated by the lumbosacral plexus. Patients with electromyographic signs of neurogenic lesions (such as diabetic neuropathy) and those with concomitant obliterative endarteritis of the lower limbs with intermittent claudication classified as grade II to IV were excluded from the study. Before the procedure, the affected skin area was disinfected, and local anesthesia was administered using ethyl chloride.

Infiltrations were performed by trained spinal interventional neuroradiologists.

A preliminary low-dose CT scan was obtained for correct centration of the level of interest to be treated. The injection site at the skin level was then identified, and a subsequent measurement of the distance to the conjugation foramen or facet joints was carried out in relation to the pathology treated.

**Figure 4:**
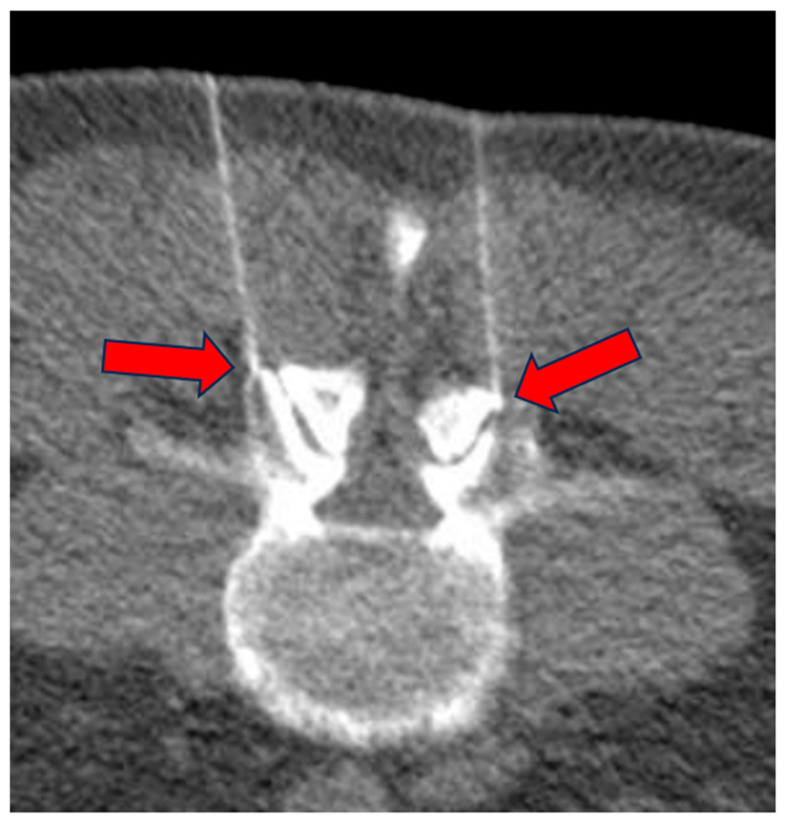
Low-dose CT scan for correct positioning of the needles at the facet joint level (arrows).

Typically, a 9 cm 22 G needle was used, but in some cases longer needles (up to 15 cm) were used depending on the patient’s anatomy. Another CT scan was then performed to confirm accurate needle placement (Figure 4).

**Figure 5:**
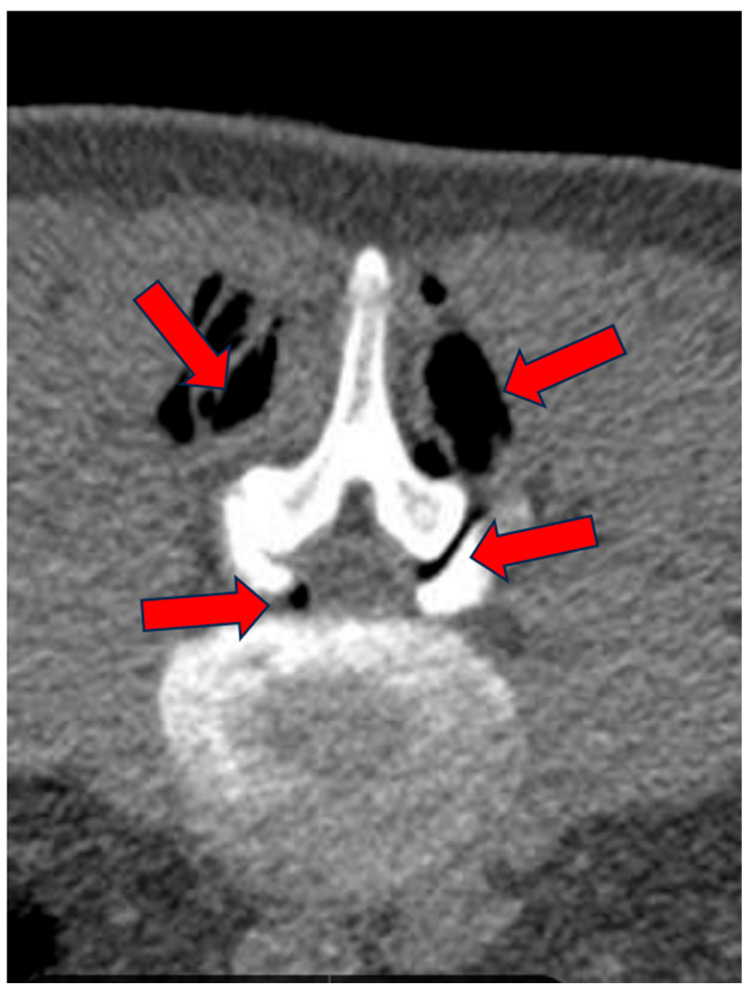
Low-dose CT scan for proper distribution of the gas mixture after infiltration (arrows).

The procedure involved injecting 3 cc of the O_2_-O_3_ gas mixture at a concentration of 25 μg/mL, followed by retracting the needle a few millimeters to inject another 5 cc of the mixture to treat the region surrounding the facet joint (Figure 5). This technique is well established and widely documented in the international literature, e.g., [20,21,22,23]. For patients suffering from facet syndrome, the infiltration was performed both at the level of the facet joints and the intervertebral foramina, while for patients with disc-related pathology, the treatment was limited to intraforaminal–periganglionic injections, in line with guidelines reported in literature, e.g., [20,21,22,23,26,30,33].

Up to three deep periganglionic–paravertebral infiltrations were performed for patients with multilevel disease.

In patients with facet syndrome, the O_2_-O_3_ mixture was injected into the joints when possible or immediately around the joint capsule when direct access to the intervertebral space was not feasible (due to osteophytes, asymmetric facet joints, or specific shapes of the joint aperture).

After the injection, a further CT check of the correct distribution of the gas mixture was performed (Figure 5).

Treatments were performed using equipment equipped with a photometric detector that monitored the O_2_-O_3_ concentration in the gas mixture. All materials used were sterile and single-use.

The oral administration of ALA + PEA + myrrh has been prescribed for a duration of 30 days for each patient, starting on the day following the initial infiltration of O_2_-O_3_.

All patients were scheduled for follow-up evaluations at one month and one year following the treatment, using a modified version of McNab’s method (Table 1).

Additionally, each patient was administered the Visual Analog Scale (VAS) to assess pain intensity. The VAS is a psychometric response scale, usually used in questionnaires to measure a characteristic or attitude that ranges across a continuum of values, helping to quantify the intensity of pain. Patients mark a point on a line (typically 10 cm long) that represents their pain level, where 0 cm indicates “no pain” and 10 cm indicates “worst pain imaginable”. The VAS was administered before the treatment and during the one-month and the one-year follow ups to evaluate changes in pain perception over time.

## 3. Results

All 276 patients in our observational study were initially (T0) assessed using the VAS only at enrollment.

Before treatment, the VAS indicated a mean score of 8.17 (±0.55) among patients, reflecting significant levels of low back pain (Table 2).

At the 1-month follow-up (T1), all 276 patients were reassessed using the VAS and the modified McNab method. According to the modified McNab criteria, 91 patients (32.94%) reported a marked improvement in clinical symptoms, with almost the complete disappearance of low back pain. Additionally, 148 patients (53.65%) were satisfied with the treatment but experienced only a partial reduction in pain. In contrast, treatment provided little or no benefit for 37 patients (13.41%) (Table 3). The results from the VAS at T1 indicated a mean score of 2.81 (±0.83) (Table 4).

**Figure 6:**
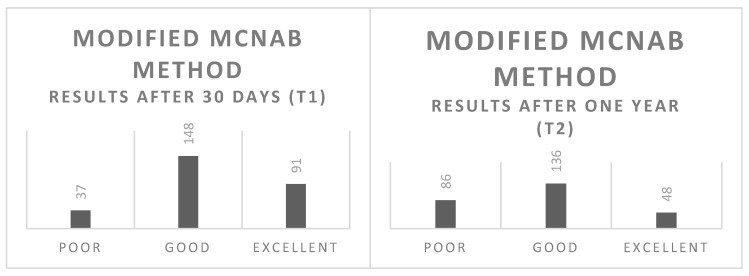
Visual representation of the results at one month (T1) and one year (T2) based on the modified McNab method. This figure illustrates the distribution of patient outcomes, highlighting the changes in quality of life and treatment efficacy over the follow-up periods.

**Figure 7:**
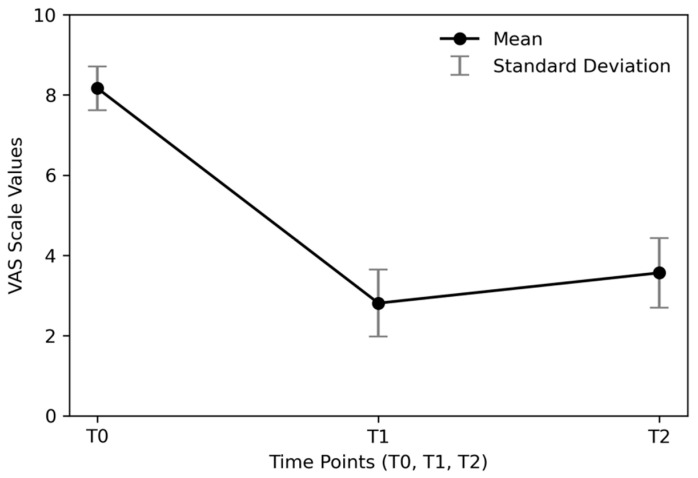
Visual representation of the distribution of results from the Visual Analog Scale (VAS) at T0, T1, and T2. This figure displays the variations in patient-reported outcomes over time, illustrating the changes in pain levels and overall satisfaction with treatment.

A one-year follow-up (T2) was conducted for 270 patients, as six had died from natural causes in the meantime. According to the modified McNab method, cumulatively, 68.15% (n = 184) reported positive outcomes. Specifically, 48 patients (17.78%) reported an excellent quality of life, with an almost complete disappearance of low back pain. The number of patients experiencing a good benefit from treatment was 136 (50.37%). Treatment was deemed mediocre or poor in 86 patients (31.85%) (Table 5). The results from the VAS at T2 indicated a mean score of 3.57 (±0.86) (Table 6; Figure 6 and Figure 7).

At the one-year follow-up, none of the patients included in our study reported undergoing additional treatments.

The analysis of the McNab classification showed substantial stability of the results between the initial evaluation (T1) and the subsequent one (T2). Although the Chi-square test (X^2^ = 7.954, *p* = 0.093) did not highlight a statistically significant difference in the distribution of the categories between T1 and T2, it is important to note that the observed values suggest a trend towards improvement or maintenance of the functional status over time. This indicates that, overall, the treatment contributed to preserving (or improving in many cases) the clinical conditions of the patients, demonstrating stability and the absence of significant worsening.

In regards to the analysis of VAS scores, given that the Shapiro–Wilk test indicated non-normal distributions (*p* < 0.001 for VAS_T0 and VAS_T1), we used non-parametric tests for pairwise comparisons. Specifically, the Wilcoxon signed-rank test was applied to compare VAS scores between time points:

- VAS_T0 vs. VAS_T1: Wilcoxon signed-rank test, *p* < 0.001
- VAS_T1 vs. VAS_T2: Wilcoxon signed-rank test, *p* < 0.001
- VAS_T0 vs. VAS_T2: Wilcoxon signed-rank test, *p* < 0.001

Additionally, we conducted a repeated-measures ANOVA to assess overall differences across time points. The results (F = 4007.09, *p* < 0.001) confirmed a statistically significant change in VAS scores over time.

To better assess the clinical significance of VAS score changes, confidence intervals for the mean VAS scores at each time point are reported:

- VAS_T0: 8.102—8.231
- VAS_T1: 2.710—2.906
- VAS_T2: 3.460—3.666

The large reduction in VAS scores from baseline to follow-ups suggests clinically meaningful improvement, which is further supported by the statistical significance of pairwise comparisons.

These findings are noteworthy when compared to the results of a previous trial involving 127 elderly patients treated exclusively with CT-guided O_2_-O_3_ therapy. In that trial, 60.6% of the sample reported excellent to good outcomes, while 39.4% reported poor results [29].

## 4. Discussion

In this observational study, we evaluated the results obtained with the combined treatment of O_2_-O_3_ therapy under CT guidance associated with oral treatment with ALA + PEA + myrrh in 276 patients monitored one month and one year after the end of therapy using both the VAS questionnaire and the modified McNab method. In the literature, studies with clinical follow-up of the efficacy of O_2_-O_3_ treatment alone report data of variable efficacy between 75% and 90% [21,22,23,24,25,26,27,28,29,30,31,32,33,34] in samples of patients with a variable age, with the percentage of therapeutic success that tends to decrease considering only elderly patients reporting success rates closer to 60%-70% [29].

In this regard, we believe that the paravertebral administration of O_2_-O_3_ in CT-guided mode guarantees perfect control of the needle path. The possibility of curative O_2_-O_3_ is high in this regard for the improvement of local circulation with eutrophication near the nerve root, compressed and affected by muscle spasms. It can normalize the level of cytokines and prostaglandins with anti-inflammatory and analgesic action, increasing the production of superoxide dismutase (SOD) while minimizing oxidizing reagents (ROS). Finally, the close proximity to the herniated/protruded material causes accelerated dehydration or destruction of non-vascularized tissue, justifying the good final result [19,20,36,37,44].

In elderly patients, osteoarthritic degeneration of the facet joints initially occurs in the cartilage, leading to a subsequent narrowing of the joint space and increased blood vessel formation, with several inflammatory mediators being involved. In the later stages of the condition, this process progresses to joint hypertrophy, the formation of osteophytes, and continuous thickening of the subchondral bone, which ultimately results in sclerosis and erosion [1,2,3,4,5,6,7,8,9,10,11,12,13,14,15,16,17,18].

Research has shown a rise in the infiltration of immune cells and pro-inflammatory cytokines (such as TNF-α, IL-1β, IL-6, and prostaglandins), along with enzymes that contribute to cartilage breakdown. Additionally, there is an upregulation of anti-inflammatory cytokines and inhibitors of these enzymes, as part of an effort to stimulate a reparative response. Angiogenic factors are also present, further promoting inflammation by increasing the local influx of inflammatory cells and stimulating neurogenesis.

Moreover, the development and worsening of osteoarthritis are linked to an increase in oxidative stress and an overproduction of reactive oxygen species (ROS). In healthy cartilage, chondrocytes produce minimal amounts of ROS, maintaining a balance with the body’s natural antioxidant systems, which helps sustain cartilage homeostasis, control the aging and apoptosis of chondrocytes, and regulate extracellular matrix synthesis. However, in osteoarthritic cartilage, excessive ROS are generated due to heightened mechanical stress, variations in oxygen pressure, and the presence of immunomodulatory mediators, while antioxidant enzymes are notably reduced. This increase in oxidative stress leads to the premature aging and death of chondrocytes and synoviocytes. Additionally, it activates NF-kB, which amplifies the production of pro-inflammatory factors.

O_2_-O_3_ therapy functions through several mechanisms:

ο Activation of cellular metabolism;
ο Reduction in the production of pro-inflammatory cytokines and prostaglandins;
ο Boosting of immunosuppressive cytokines;
ο Reduction in oxidative stress caused by chronic oxidative stress;
ο Enhancement of oxygen delivery to tissues [19,20,21,22,23,24,25,26,27,28,29,30,31,32,33,34,35].

In the context of osteoarthritis, O_2_-O_3_ therapy acts as a modulator of the inflammatory response, promoting the production of TNF-β while reducing levels of pro-inflammatory cytokines, such as TNF-α and IL-8. Furthermore, it influences the regulation of the NF-kB pathway, which is responsible for increasing pro-inflammatory cytokine production, contributing to impaired extracellular matrix synthesis, cartilage damage, changes to the cartilage matrix, and apoptosis. The NF-kB pathway is directly activated by ROS or by pro-inflammatory mediators like TNF-α. O_2_-O_3_ therapy directly diminishes ROS production or indirectly inhibits TNF-α, thus blocking the NF-kB pathway [19,20,21,22,23,24,25,26,27,28,29,30,31,32,33,34,35].

Administering low doses of O_2_-O_3_ also stimulates the body’s response by encouraging the production of antioxidant enzymes, helping the body adapt to chronic oxidative stress and restore the redox balance. Finally, it enhances the efficient use of oxygen in the mitochondrial respiratory chain, stimulating glycolysis in damaged cells and thereby preventing cell death [19,20,36,37,44].

The ease of performing the method and the complete control of the infiltration via CT allow for the proposal of the technique of CT-guided O_2_-O_3_ therapy as a valid therapeutic choice among conservative treatments in LBP, e.g., [24,25,34]. Our findings, in line with previous literature regarding the effectiveness of O_2_-O_3_ in the elderly [29], suggest that this treatment may be effective and well-tolerated in this age group, aligning with previous observational data.

In our observational study, we documented how the association with a minimally invasive therapy such as O_2_-O_3_ therapy under CT, combined with the oral administration of 800 mg/day of ALA, 600 mg/day of PEA, and 200 mg of myrrh, allows for a substantial increase in the percentage of clinical improvement in these patients. This combination appears to be a promising therapeutic approach, associated with improved clinical outcomes and symptom control in the studied cohort. This is particularly attributable to the synergistic action of ALA, PEA, and myrrh, which effectively alleviates neuropathic symptoms [45,46,47,48,49,50,51,52,53,54,55,56,57,58].

By comparing the results obtained with an observational study on 318 patients afflicted exclusively by sciatic pain due to herniated discs (median age: 47.2), it is possible to deduce how, although the percentage of therapeutic success in elderly patients is slightly lower in relation to the age-related degenerative picture of the lumbar spine, the data reported in this study must be considered extremely satisfactory in relation to the sample of subjects and the type of pathology treated [59].

Additional support for these findings comes from a previous one-year clinical trial of O_2_-O_3_ therapy alone involving 129 elderly patients enrolled at baseline. After one year, 127 patients completed the follow-up, as two had died. In that study, excellent-to-good outcomes were observed in 60.6% of the cases, while 39.4% were classified as poor. Notably, the addition of this therapeutic approach yielded excellent-to-good results in 68.15% of patients, with poor outcomes in only 31.85%.

As this is a one-year observational study, a future objective will be to evaluate patients for pain recurrence beyond the timeframe of one year.

On the basis of the results obtained in this study on a sample of 276 patients, we therefore believe that this additional therapeutic option can be offered to elderly patients suffering from LBP.

## 5. Conclusions

In conclusion, our study highlights the promising therapeutic effects of combined O_2_-O_3_ therapy under CT guidance with the oral administration of ALA, PEA, and myrrh in elderly patients suffering from chronic low back pain. The significant reductions in pain levels, as evidenced by the VAS scores and the modified McNab method, demonstrate the efficacy of this dual therapeutic approach.

Given the aging population and the increasing prevalence of low back pain among the elderly, it is imperative to explore and implement effective, safe, and minimally invasive treatment options. The findings of our study support the use of this combined therapy as a valuable option that can enhance clinical outcomes, improve quality of life, and reduce the socio-health burden associated with chronic pain in this vulnerable population.

Further research with larger sample sizes and longer follow-up periods is warranted to solidify these findings and explore the long-term benefits and potential mechanisms of action underlying this treatment strategy. Ultimately, our results enrich the existing body of knowledge regarding pain management in the elderly and pave the way for more personalized and effective treatment modalities moving forward.

## Figures and Tables

**Table 1:** Modified McNab Method.

Items	Explanation
Excellent	Resolution of pain and return to normal daily activities that were performed prior to the onset of pain
Good or Satisfactory	Pain reduction greater than 50%.
Fair or Poor	Partial pain reduction of less than 50%.

**Table 2:** VAS scale results before treatment (T0).

Instrument	Mean Score	Standard Deviation	Range of Scores
VAS Scale	8.17	0.55	7–9

**Table 3:** McNab test results after 30 days (T1).

Instrument	Excellent	Good	Poor
Modified McNab method	91 (32.94%)	148 (53.65%)	37 (13.41%)

**Table 4:** VAS scale results after 30 days (T1).

Instrument	Mean Score	Standard Deviation	Range of Scores
VAS Scale	2.81	0.83	2–5

**Table 5:** McNab test results after one year (T2).

Instrument	Excellent	Good	Poor
Modified McNab method	48 (17.78%)	136 (50.37%)	86 (31.85%)

**Table 6:** VAS scale results after one year (T2).

Instrument	Mean Score	Standard Deviation	Range of Scores
VAS Scale	3.57	0.86	2–7

## Data Availability

The original contributions presented in this study are included in the article. Further inquiries can be directed to the corresponding author(s).
